# Attrition between lines of therapy and real-world outcomes of patients with HER2-positive metastatic breast cancer in Europe: a cohort study leveraging electronic medical records

**DOI:** 10.1007/s10549-024-07506-4

**Published:** 2024-10-20

**Authors:** Paul Cottu, Sue Cheeseman, Peter Hall, Achim Wöckel, Christian W. Scholz, Emilio Bria, Armando Orlandi, Nuria Ribelles, Mahéva Vallet, Nicolas Niklas, Catherine Hogg, Shivani Aggarwal, Joana Moreira, Markus Lucerna, Simon M. Collin, Amanda Logue, Gráinne H. Long

**Affiliations:** 1 Department of Medical Oncology, Institut Curie, Université Paris Cité, Paris, France; 2 Leeds Teaching Hospitals NHS Trust, Leeds, UK; 3 University of Edinburgh, Edinburgh, UK; 4 Edinburgh Cancer Centre, Western General Hospital, NHS Lothian, Edinburgh, UK; 5 Department of Gynecology, University Hospital Würzburg, Würzburg, Germany; 6 Department of Hematology and Oncology, Vivantes Klinikum Am Urban, Berlin, Germany; 7 Unit of Oncology, Comprehensive Cancer Center, Fondazione Policlinico Universitario Agostino Gemelli IRCCS, Università Cattolica del Sacro Cuore, Rome, Italy; 8 Ospedale Isola Tiberina – Gemelli Isola, Rome, Italy; 9 Department of Medical Oncology, Hospital Universitario Virgen de La Victoria, IBIMA, Málaga, Spain; 10 IQVIA, Frankfurt, Germany; 11 IQVIA, London, UK; 12 Landmark Science, Los Angeles, CA USA; 13 IQVIA, Lisbon, Portugal; 14 Daiichi Sankyo Europe GmbH, Munich, Germany; 15 Oncology Outcomes Research, Global Medical Affairs, Oncology Business Unit, AstraZeneca, Cambridge, UK; 16 Medical Communications and Information, Global Medical Affairs, Oncology Business Unit, AstraZeneca, Cambridge, UK

**Keywords:** Breast neoplasm, Neoplasm metastasis, ERBB2 protein, HER2, Real-world data, Attrition

## Abstract

**Purpose:**

To characterize real-world attrition rates across first-line (1L) to third-line (3L) therapies in patients with HER2-positive (HER2 +) metastatic breast cancer (mBC) receiving routine care in seven hospital systems across Europe (France, Germany, Italy, Spain, and the UK).

**Methods:**

This retrospective, observational, multi-country, cohort study collected electronic medical record data from women aged ≥ 18 years diagnosed with HER2 + mBC from 2017–2021. The primary endpoint was attrition rate (the proportion of patients receiving a line of therapy [LOT] with no further evidence of subsequent LOTs). Key additional endpoints included treatment patterns, real-world time to treatment discontinuation (TTD), and time to next treatment (TTNT).

**Results:**

29.6% (95% confidence interval [CI] 25.0–34.6) and 34.2% (95% CI 27.5–41.5) of treated patients with HER2 + mBC had no further evidence of treatment beyond 1L and second-line (2L) therapy, respectively. Attrition was primarily owing to death, move to end-of-life palliative care, loss to follow up, and “other” reasons. Treatment patterns were generally aligned with clinical guidelines. Decreases in TTD (12.1 months [95% CI 10.4–14.5] for 1L, 8.9 months [95% CI 7.3–11.9] for 2L, 6.4 months [95% CI 5.2–8.9] for 3L) and TTNT (15.4 months [95% CI 13.6–20.6] for 1L, 13.5 months [95% CI 10.8–19.4] for 2L) were observed with each subsequent LOT.

**Conclusion:**

Results unveil a large proportion of patients who do not benefit from state-of-the-art subsequent LOT, and suggest diminishing effectiveness with each subsequent LOT.

**Supplementary Information:**

The online version contains supplementary material available at 10.1007/s10549-024-07506-4.

## Introduction

Breast cancer (BC) is the most common malignancy among women in European Union countries, with a total of 374,800 cases in 2022, accounting for 29.4% of all cancer diagnoses [1]. Approximately 20% of all BC cases have HER2-positive (HER2 +) disease [2, 3], an aggressive subtype associated with poorer outcomes and higher mortality rates than HER2-negative disease [4, 5].

The introduction of HER2-directed therapies has contributed to improved clinical outcomes for patients with HER2 + metastatic breast cancer (mBC) compared with previous standard-of-care therapies [4, 6–10]; however, there is substantial drug-sequencing heterogeneity [11, 12]. Online resource 1 summarizes the HER2-directed agents approved for use in patients with HER2 + mBC. Despite the introduction of HER2-directed therapies, patients frequently experience disease relapse and require subsequent lines of therapy (LOTs) [9, 13].

Each subsequent LOT for patients with HER2 + mBC is associated with shorter treatment durations and poorer efficacy than previous LOTs [14]. Receiving the optimal targeted therapy in the earliest indicated setting is important to maximize the likelihood of durable clinical benefit [15, 16]. If patients do not receive guideline-directed care in the earliest indicated setting, the benefit gained from subsequent LOTs may be diminished or they may not receive a subsequent LOT [17–19]. There is a paucity of literature reporting attrition rates among patients with HER2 + mBC, with limited real-world studies conducted across European countries showing the proportion of patients with HER2 + mBC receiving a subsequent LOT decreasing from first-line (1L) to fourth-line treatment [9, 20]. As more therapies become available, understanding treatment patterns and sequencing may help guide treatment decision-making and inform the optimal treatment paradigm for patients with HER2 + mBC [11].

 The study aim was to characterize real-world attrition rates across 1L to third-line (3L) therapies in patients diagnosed with HER2 + mBC between 2017 and 2021 who received routine care in France, Germany, Italy, Spain, and the UK.

## Methods

This was a retrospective, observational, multi-country, multicenter, cohort study describing attrition rates among patients with HER2 + mBC using electronic medical record (EMR) data from seven hospital groups in five European countries. Online resource 2 shows the study design.

### Data sources

Eligible patients were identified from IQVIA’s Oncology Evidence Network centers within European countries (one hospital in France, two hospital groups in Germany, one hospital in Italy, one hospital group in Spain, and two hospital groups in the UK). Centers were selected based on their extensive experience in treating patients with BC and the feasibility of gathering EMR data. Details about each center are available in Online resource 3.

BC diagnosis, metastatic status, hormone receptor (HR), and HER2 status were derived from diagnosis codes, tumor staging, and diagnostic tests reported in each center’s EMRs, along with age and sex at birth. Patients had histologically confirmed BC with metastases and were identified using the International Classification of Diseases, 10th Revision, and Clinical Modification codes within the malignant neoplasm of breast hierarchy. The Union for International Cancer Control staging was used to determine metastatic disease.

Structured EMR data and manually abstracted unstructured data (patient medical charts) were curated, cleaned, and quality checked according to center-specific standards. Data were harmonized across centers using a common data model and were collected for all systemic treatments. The information captured for each LOT was based on treatment start and end dates, as well as individual drug(s), drug regimen(s), and drug class(es) contributing to each LOT.

### Study population

Women were eligible for study inclusion if they had histologically confirmed BC with initial metastatic (Stage IV) or early-stage disease that later became metastatic between January 01, 2017, and June 30, 2021, and were ≥ 18 years old at the time of metastatic disease. Patients were required to have a locally determined HER2 immunohistochemistry value of 3 + or a ratio of ≥ 2.0 by in situ hybridization (ISH) or fluorescence ISH closest to the date of metastasis. Participation in any interventional clinical trial on or after the date of metastasis, presence of other co-malignancies within 1 year prior to the metastasis date, except non-melanoma skin cancer and in-situ or benign neoplasms, the capture of incomplete treatment pathway information, or opting out of re-use of data, were exclusionary.

Patients were followed up from treatment initiation (LOT initiation/index date), until the outcome of interest, a censoring event, or the end of the study period (June 30, 2022), whichever occurred first. Based on precision estimates for the primary endpoint and a preliminary feasibility survey of eligible patient numbers at each center, a target of 100 patients per country was set.

### Endpoints

**Fig. 1:**
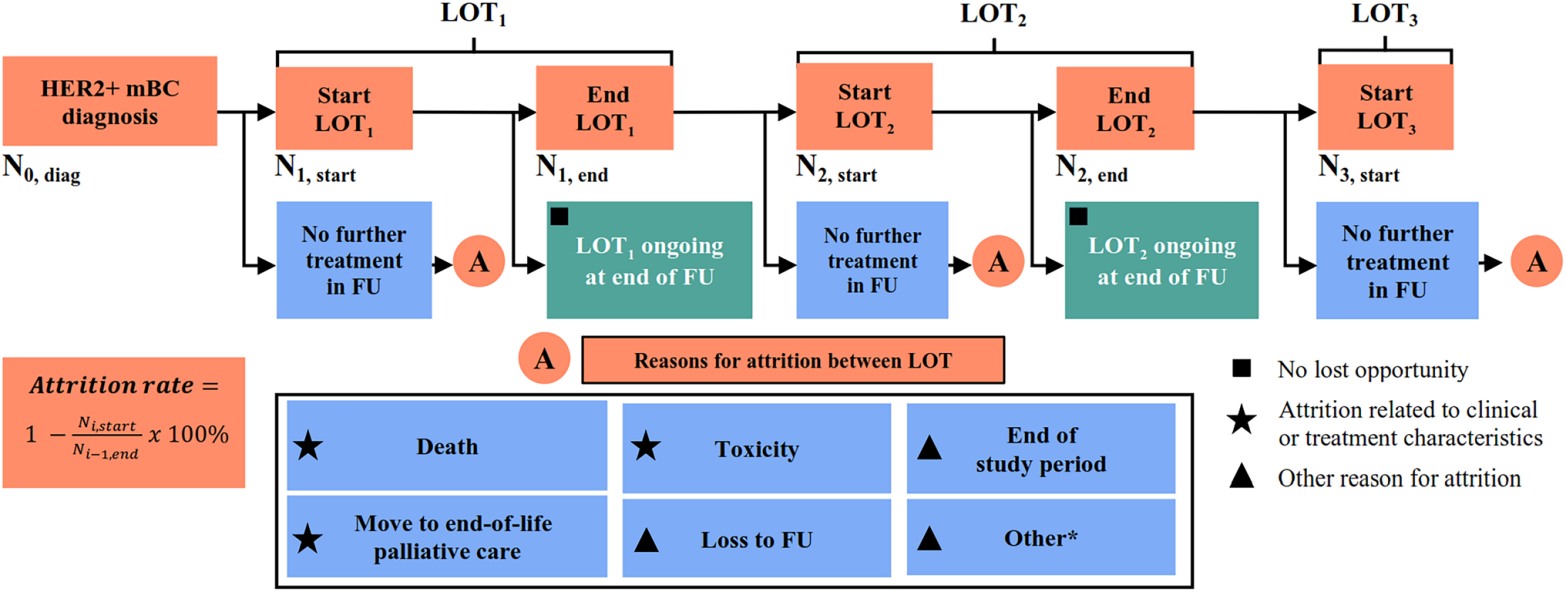
Attrition algorithm. *A* attrition, *FU* follow up, *HER2* + human epidermal growth factor receptor 2-positive, *LOT* line of therapy, *mBC* metastatic breast cancer, *N* number of patients

The primary endpoint was attrition rate, defined as the proportion of patients receiving a LOT with no further evidence in EMRs of subsequent LOTs, calculated from 1L to second-line (2L) and 2L to 3L. Figure 1 shows the equation used to calculate attrition rates.

LOTs were derived algorithmically referencing the European Society for Medical Oncology (ESMO) guidelines [21]. The treatment holiday window was determined based on input from IQVIA’s Medical Director; re-initiation of the same drug within a treatment gap of 365 days from the last treatment end date for that drug did not advance the LOT. The LOT algorithm was validated manually for a sample of *n* = 100 patients from France, Germany, and the UK, and by two sensitivity analyses (Online resource 4). The final LOT algorithm was applied to each oncology center’s data to ensure consistency of interpretation.

Documented reasons for attrition included death or move to end-of-life (EOL) palliative care (within 30 days of end of LOT), discontinuation owing to toxicity, end of study period, loss to follow up (FU), or “other.” Reasons were classified as “other” if they did not meet any of the categories defined above, or if death or move to EOL palliative care occurred > 30 days after treatment discontinuation. The 30-day window was selected following discussions with a Senior Epidemiologist and IQVIA’s Medical Director to maximize the chance of identifying true drivers of treatment cessation, as events including death, move to EOL palliative care, and toxicities are less likely to be attributable to treatment cessation beyond this window. In the very rare occasion when more than one reason for attrition occurred within 30 days of treatment discontinuation, the most relevant, severe, and/or terminal reason (death) was recorded as the reason for attrition.

Key secondary analyses included characterizing: (1) time to discontinuation (TTD, the length of time from the index date to subsequent LOT initiation, death, censoring at the last known activity date at the oncology center or end of the study period, whichever came first) for 1L to 3L, (2) demographic and clinical characteristics, and (3) treatment patterns. Drugs were categorized by treatment class, including HER2-directed therapies, endocrine therapy, chemotherapy (e.g., taxanes, platinum-based compounds, others), cyclin-dependent-kinase 4/6 inhibitors (CDK4/6i), endocrine therapy + CDK4/6i, and other. Treatments were flagged as maintenance therapy if they could be categorically identified as such; therefore, if pertuzumab and/or trastuzumab were used in 1L therapy, patients were not flagged for maintenance owing to the inability to identify whether these drugs were administered as part of 1L or maintenance therapy.

Exploratory analyses included characterizing: (1) time to next treatment (TTNT, the time from the LOT start date to subsequent LOT initiation, death, censoring at the last known activity date at the oncology center or end of study, whichever occurred first) for 1L to 2L, (2) progression-free survival (PFS, the time from LOT start date to a progression or death event, censoring at the subsequent LOT initiation, last known activity date at the oncology center, or end of study period, whichever occurred first) for 1L to 3L, and (3) factors associated with receiving a subsequent LOT identified by statistical models.

### Analytical plan

Descriptive statistics were used to summarize treatment patterns and demographic and clinical characteristics. Continuous variables were outlined by providing the number of observations, mean and standard deviation, median, interquartile ranges (IQRs), and overall ranges. Categorical variables were summarized by providing counts and proportions, with missing data considered a separate category. Point estimates and 95% confidence intervals (CIs) are presented. For time-to-event outcomes, median time to event, 95% CIs, and 6-, 12- and 18-month probabilities were estimated. Univariate and multivariable Cox proportional hazard regression models were fitted to identify variables independently associated with TTNT for 1L and 2L therapy; variables with univariate *P* < 0.1 were carried forward to multivariable models. Online resource 5 notes the packages and workstreams used by the statistical tool R v4.1.3.

## Results

### Patient demographics and clinical characteristics

Data from 496 women from across seven hospital groups in five European countries were included. Patient demographics and clinical characteristics are summarized in Table 1 (see Online resource 6 for data stratified by country and HR status). Median duration of FU was 41.1 months (IQR 22.4–53.0).

**Table 1:** Patient demographics and clinical characteristics

	Overall *N* = 496
Median age at initial diagnosis (IQR), years	56.0 (47.0–70.0)
Median age at mBC diagnosis (IQR), years	59.0 (49.0–72.0)
Median BMI (IQR), kg/m^2^	25.7 (22.5–29.7)
Postmenopausal status at mBC diagnosis, *n* (%)	293 (59.1)
Current smoker,* n* (%)	75 (15.1)
Primary tumor type, *n* (%)	
Invasive ductal carcinoma	424 (85.5)
Invasive lobular carcinoma	22 (4.4)
Other	29 (5.9)
Unknown	17 (3.4)
Missing	4 (0.8)
HR status, *n* (%)	
HR +	328 (66.1)
HR −	163 (32.9)
HR missing	5 (1.0)
Metastatic sites at BC diagnosis, *n*** (**%)	
< 4	360 (72.6)
≥ 4	136 (27.4)
Metastatic location, *n*** (**%)^a^	
Local/breast	146 (29.4)
Brain	151 (30.4)
Bone	285 (57.5)
Lung	193 (38.9)
Liver	229 (46.2)
Lymph nodes	258 (52.0)
Other	111 (22.4)
Grading, *n* (%)	
Grade 1	3 (0.6)
Grade 2	157 (31.7)
Grade 3	289 (58.3)
Unknown	42 (8.5)
Missing	5 (1.0)
Stage at initial BC diagnosis, *n*** (**%)	
0/I/II/III	169 (34.1)
IV	302 (60.9)
Unknown	18 (3.6)
Missing	7 (1.4)
Total number of LOTs per patient, *n*** (**%)^b^	
0	34 (6.9)
1	208 (41.9)
2	131 (26.4)
3	61 (12.3)
Median duration of FU (IQR), months	41.1 (22.4, 52.8)

*BMI* body mass index, *FU* follow up, *HR* hormone receptor, *IQR* interquartile range, *LOT* line of therapy, *mBC* metastatic breast cancer

^a^Patients may belong to > 1 category

^b^*n* = 62 patients received ≥ 4 LOTs per patient

Median age at initial BC and mBC diagnosis was 56 (IQR 47–70) and 59 (IQR 49–72) years, respectively. Overall, 60.9% (*n* = 302/496) of patients had Stage IV de-novo disease at initial BC diagnosis. Most patients were postmenopausal at mBC diagnosis (59.1%, *n* = 293); 15.1% (*n* = 75/496) were current smokers. Invasive ductal carcinoma was reported in 85.5% of patients (*n* = 424/496). In total, 328 (66.1%) patients were HR–positive, 163 (32.9%) HR–negative, and HR status was missing for 5 (1.0%) patients. Stage IV disease was the most common stage at initial diagnosis (60.9% [*n* = 302/496]). Overall, 77.4% of patients (*n* = 384/496) had ≥ 2 metastatic sites at BC diagnosis; bone (57.5%, *n* = 285/496) and lymph node metastases (52.0%, *n* = 258/496) were the most common; brain metastases occurred in 30.4% of patients (*n* = 151/496).

### Treatment patterns

**Fig. 2:**
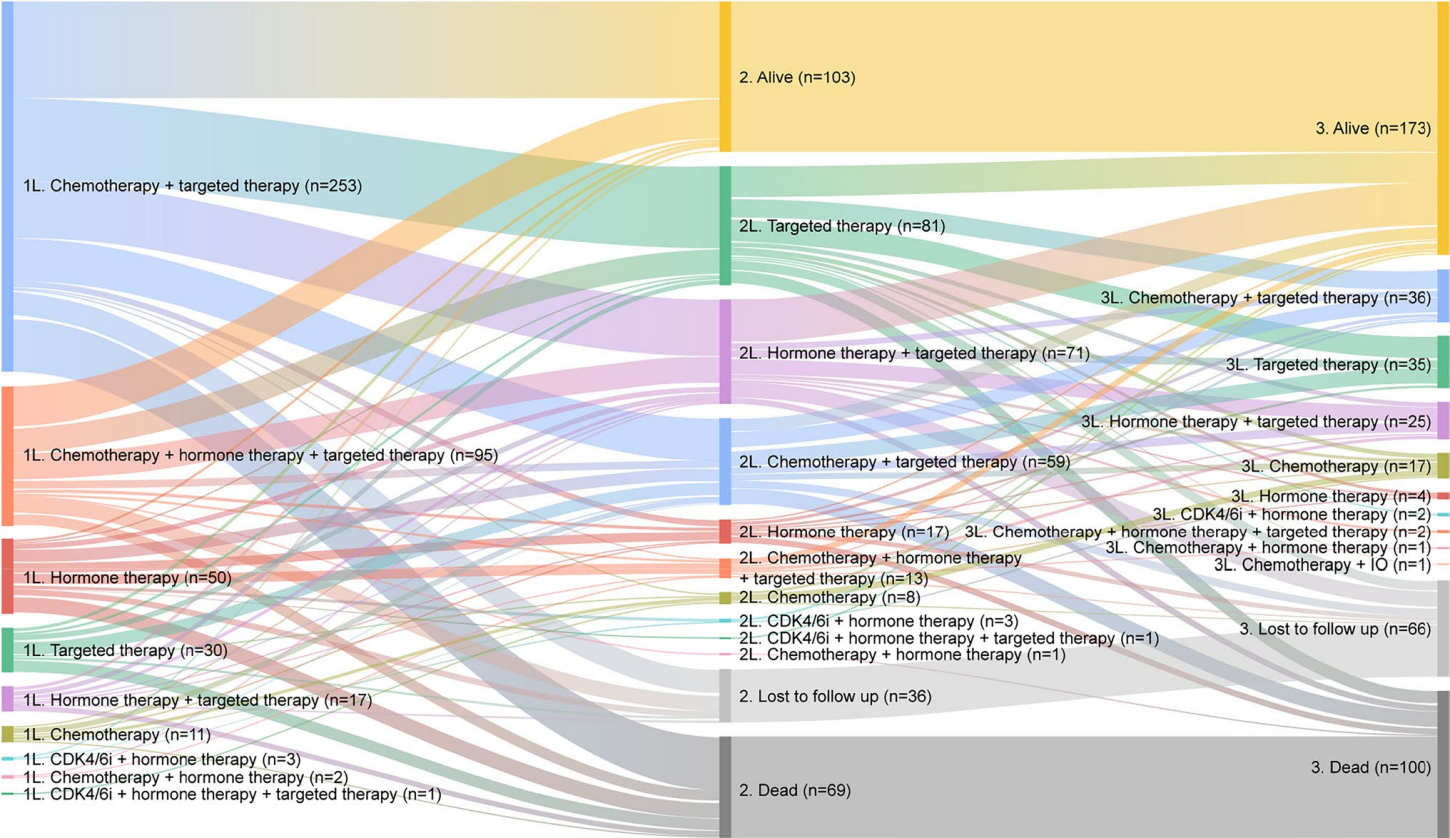
Treatment patterns on a regimen level from 1 to 3L. *1L* first line, *2L* second line, *3L* third line, *CDK4/6i* cyclin-dependent kinase 4 and 6 inhibitor, *IO* immunotherapy

Overall, 93.1% of patients (*n* = 462/496) received at least one LOT in the metastatic setting; 51.2% of patients (*n* = 254/496) started 2L therapy and 24.8% (*n* = 123/496) patients started 3L therapy. In 1L 54.8% (*n* = 253/462) of patients received HER2-directed therapy in combination with chemotherapy, in 2L 30.3% (*n* = 77/254) of patients received a HER2-directed antibody–drug conjugate, and in 3L 29.3% (*n* = 36/123) of patients received HER2-directed therapy in combination with chemotherapy (Fig. 2). The most common regimens in 1L contained pertuzumab and trastuzumab in combination (65.2%, *n* = 301/462), at 2L the most common regimens were trastuzumab emtansine-containing therapies (37.4%, *n* = 95/254), and at 3L the most common regimens had trastuzumab as the only HER2-directed therapy (28.5%, *n* = 35/123). Median duration of therapy was 8.0 months (IQR 3.9–16.2) for 1L, 5.4 months (IQR 2.6–11.9) for 2L, and 4.9 months (IQR 2.5–9.2) for 3L; median TTD was 12.1 months (95% CI 10.4–14.5) for 1L, 8.9 months (95% CI 7.3–11.9) for 2L, and 6.4 months (95% CI 5.2–8.9) for 3L. The proportion of patients with ongoing 1L or 2L treatment at end of study was 22.3% (*n* = 103/462) and 27.2% (*n* = 69/254), respectively.

### Attrition rates across LOT

**Fig. 3:**
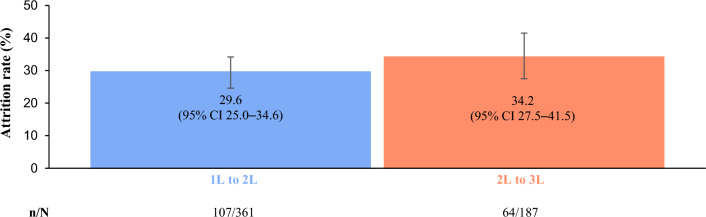
Attrition rates from 1L to 2L and 2L to 3L in the overall cohort. *1L* first line, *2L* second line, *3L* third line, *CI* confidence interval

Overall attrition rates from 1 to 2L and 2L to 3L were 29.6% (*n* = 107/361 [95% CI 25.0–34.6]) and 34.2% (*n* = 64/187 [95% CI 27.5–41.5]), respectively (Table 2, Fig. 3). Attrition rate data stratified by country and HR status are reported in Online resource 7. Death (37.4%, *n* = 40) accounted for the majority of 1L to 2L attrition, followed by “other” (29.9%, *n* = 32) and EOL palliative care (15.0%, *n* = 16); “other” (40.6%, *n* = 26) and death (25.0%, *n* = 16) were the most common reasons for 2L to 3L attrition (Table 3).

**Table 2:** Overall attrition rates between LOT

	Overall *N* = 496
1L to 2L	
Start of 1L, *n*	462
Completed 1L,* n*	361
Attrition rate, % (95% CI)	29.6 (25.0–34.6)
2L to 3L	
Start of 2L, *n*	254
Completed 2L, *n*	187
Attrition rate, % (95% CI)	34.2 (27.5–41.5)

*1L* first line, *2L* second line, *3L* third line, *CI* confidence interval, *LOT* line of therapy

**Table 3:** Overall reasons for attrition between LOT

	*n*	Attrition rate
		% (95% CI)
1L to 2L		
Death	40	37.4 (28.2–47.3)
EOL palliative care	16	15.0 (8.8–23.1)
Toxicity	2	1.9 (0.2–6.6)
Loss to FU	15	14.0 (8.1–22.1)
End of study period	2	1.9 (0.2–6.6)
Other	32	29.9 (21.4–39.5)
2L to 3L		
Death	16	25.0 (15.0–37.4)
EOL palliative care	6	9.4 (3.5–19.3)
Toxicity	3	4.7 (1.0–13.1)
Loss to FU	8	12.5 (5.6–23.2)
End of study period	5	7.8 (2.6–17.3)
Other	26	40.6 (28.5–53.6)

*1L* first line, *2L* second line, *3L* third line, *CI* confidence interval, *EOL* end of life, *FU* follow up, *LOT* line of therapy

A post-hoc analysis of the “other” category identified that some patients were categorized as “other” owing to the 30-day event window in the reason for attrition algorithm (Online resource 8). When the window is extended beyond 30 days, we observed an increased proportion of patients with EOL palliative care, loss to FU, or death as the reason for attrition. Further, a minority of patients in the “other” category were identified as discontinuing treatment owing to stable disease, patient choice, and deteriorating condition.

### TTNT from 1 to 2L

**Fig. 4:**
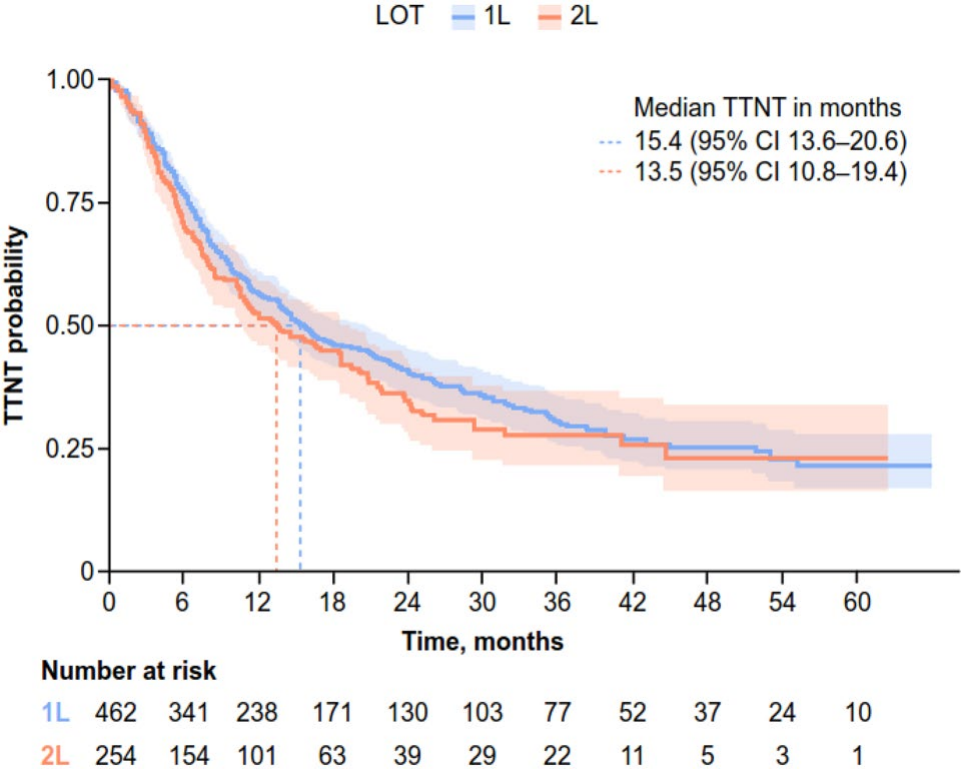
TTNT from 1 to 3L. *1L* first line, *2L* second line, *CI* confidence interval, *LOT* line of therapy, *TTNT* time to next treatment

Median TTNT was 15.4 months (95% CI 13.6–20.6) for 1L and 13.5 months (95% CI 10.8–19.4) for 2L (Fig. 4). Multivariable time-to-event models with stepwise selection identified age, number of sites of metastases at BC diagnosis, and grade as being associated with TTNT (Table 4). For 1L, patients with Grade 3 disease had a longer TTNT versus Grade ≤ 2 (hazard ratio 0.77 [95% CI 0.60–0.99; *P* < 0.043]). For 2L, premenopausal status at mBC diagnosis was associated with a longer TTNT than postmenopausal status (hazard ratio 0.66 [95% CI 0.44–0.98; *P* < 0.042]). Similarly, longer TTNT at 1L and 2L was associated with one site versus ≥ 2 sites of metastases at BC diagnosis (hazard ratio 0.73 [95% CI 0.54–0.99]; *P* < 0.045 and hazard ratio 0.48 [95% CI 0.28–0.82]; *P* < 0.007, respectively). See online resources 9–11 for 1L and 2L univariate time-to-event models, and PFS.

**Table 4:** Factors associated with TTNT^a^

		Hazard ratio (95% CI)	*P* value^b^
1L			
Age (years)	< 50	Reference	
	50– ≤ 60	0.90 (0.65–1.24)	0.509
	60– ≤ 70	0.77 (0.57–1.04)	0.087
	≥ 70	1.36 (0.89–2.06)	0.152
Number of sites of metastases at BC diagnosis	≥ 2	Reference	
	1	0.73 (0.54–0.99)	0.045
Grade	Grade 1–2	Reference	
	Grade 3	0.77 (0.60–0.99)	0.043
	Unknown	0.63 (0.39–1.02)	0.059
2L			
Number of sites of metastases at BC diagnosis	≥ 2	Reference	
	1	0.48 (0.28–0.28)	0.007
Menopausal status at mBC diagnosis	Postmenopausal	Reference	
	Premenopausal	0.66 (0.44–0.98)	0.042
	Unknown	0.52 (0.29–0.93)	0.029

*1L* first line, *2L* second line, *BC* breast cancer, *CI* confidence interval, *mBC* metastatic breast cancer, *TTNT* time to next treatment

^a^Multivariable Cox regression model using variables carried forward from Table 1 if evidence of association (*P* < 0.1) with TTNT in univariate analyses

^b^*P*-value derived from two-sided Wald test

## Discussion

In this cohort of patients with HER2 + mBC across seven hospital groups in Europe, 29.6% and 34.2% of treated patients had no further evidence of treatment beyond 1L and 2L therapy, respectively. Attrition was primarily owing to death, move to EOL palliative care, loss to FU, and “other” reasons. “Other” reasons accounted for 29.9% of 1L to 2L attrition (*n* = 32) and was the most common reason for attrition between 2L and 3L (40.6%, *n* = 26). Median treatment duration decreased with each LOT (8.0 months [IQR 3.9–16.2] for 1L, 5.4 months [IQR 2.6–11.9] for 2L, and 4.9 months [IQR 2.5–9.2] for 3L).

HER2-directed regimens are recommended for 1L and 2L treatment of patients with HER2 + mBC, regardless of HR status, per ESMO and national guidelines [21–26]. In this study 1L regimens were generally aligned with clinical guidelines; 65.2% of patients (*n* = 301/462) received regimens containing pertuzumab–trastuzumab combination therapy, reflecting the pertuzumab + trastuzumab + docetaxel regimen approved for 1L treatment of adult patients with HER2 + mBC based on results from the CLEOPATRA trial [27]. First-line median PFS in this study (18.9 months [95% CI 15.4–23.0]) was similar to median PFS in the CLEOPATRA trial (18.5 months [hazard ratio for progression or death 0.62; 95% CI 0.51–0.75, *P* < 0.001]) (Online resource 11) [6]. However, 14.5% (*n* = 67/462) of patients at 1L and 11.4% (*n* = 29/254) at 2L in our study did not receive HER2-directed therapies and were treated with chemotherapy/targeted therapies only, endocrine monotherapy, or other treatments.

Limited real-world studies in patients with HER2 + mBC in Europe have reported substantial attrition across LOTs [9, 20, 28, 29], with two studies showing an increase in attrition as patients moved from first- to later-lines, in agreement with our study [9, 20]. A study in France (*n* = 6030) reported a 52% decrease in the proportion of patients treated from 1 to 3L [9], and a study in the Netherlands (*n* = 289) estimated that 70–80%, 51–66%, and 33–54% of patients who started 1L treatment went on to start 2L, 3L and 4L treatment, respectively [20]. To our knowledge, all European real-world studies have demonstrated significant attrition across 1L to 3L [9, 20, 28, 29]. Whilst the current study reported an increasing proportion of patients with HER2 + mBC receiving a LOT with no further evidence of subsequent LOT across 1L to 3L, the German PRAEGNANT study (*n* = 776) reported less attrition from 2 to 3L (17.4%) compared with 1L to 2L (31.1%) [28]. A chart review study by Colomer et al. (undertaken in Spain, Italy, the Netherlands) reported that the proportion of patients who started a LOT, including those ongoing therapy, who did not receive subsequent antitumor treatment was substantial but similar at 1L and 2L (45% and 42%, respectively) [29]. A retrospective cohort study of patients with HER2 + mBC (N = 710) in an Italian hospital reported similar trends to the current study; median treatment duration was longer at 1L (15.3 [95% CI 13.4–17.6]) but similar at 2L (5.9 months [95% CI 5.0–8.7]) [14]. A French personalized reimbursement model database showed that ~ 30% of patients were not treated with guideline-recommended therapies in 1L, despite increased use of HER2-directed treatments during 2017–2018 versus 2011–2017 reflecting updated guidelines for patients with HER2 + mBC in Europe, [9]. This is in line with the 34.8% of patients in the current study who did not receive regimens containing pertuzumab–trastuzumab combination therapy at 1L.

In our study, 60.9% of patients had Stage IV de-novo disease at initial diagnosis. This is in line with recent real-world studies, which have reported de-novo (Stage IV) disease in 43.5‒61.9% of patients with HER2 + mBC [30–33]. The SystHERs registry study reported that patients with de-novo HER2 + mBC (*n* = 487) exhibited longer PFS and overall survival than patients with recurrent HER2 + mBC (*n* = 490) (hazard ratio 0.69 [95% CI 0.59‒0.80; *P* < 0.0001] and 0.55 [95% CI 0.44‒0.69; *P* < 0.0001], respectively) [30]. The proportion of patients with Stage IV de-novo disease at initial diagnosis in our study may have inflated TTNT and impacted the attrition rates observed, compared with settings in which the proportion of de-novo cases is substantially lower.

A decrease in TTNT was observed with each LOT (1L, 15.4 months [95% CI 13.6–20.6]; 2L, 13.5 months [95% CI 10.8–19.4]), as expected in patients with mBC. Patients with Grade 3 disease had a longer 1L TTNT versus Grade ≤ 2, which could have been driven by treatment differences associated with disease severity. Patients with ≥ 2 sites of metastases at BC diagnosis, and those with premenopausal status at mBC diagnosis, also had a longer TTNT at 2L than their peers. At 2L, median TTD was notably shorter in duration (8.9 months [95% CI 7.3–11.9]) than median TTNT (13.5 months [95% CI 10.8–19.4]). Disease burden often increases over time when patients undergo multiple LOTs, resulting in patients discontinuing treatment owing to lack of available treatment options and/or disease progression, which may contribute to the short TTD reported in this study.

At study end (June 2022), tucatinib + trastuzumab + capecitabine and T-DXd monotherapy had recently been approved for ≥ 3L treatment of patients with HER2 + unresectable/mBC (2020 and 2021, respectively) [34, 35]. T-DXd monotherapy was later approved for ≥ 2L treatment in June 2022 [36]. A small number of patients in this study received regimens containing tucatinib (at 1L *n* = 1, at 2L *n* = 1, at 3L *n* = 3) or T-DXd (at 1L *n* = 0, at 2L *n* = 1, at 3L *n* = 9). The SONABRE study reported improved 1L PFS and overall survival in patients diagnosed with de-novo HER2 + mBC between 2013 and 2017 compared with those diagnosed between 2008 and 2012, likely related to increased use of HER2-directed therapies and the approval of pertuzumab + trastuzumab + docetaxel as a 1L treatment in 2013 [7, 37]. Had the end date of the current study been extended to capture more patients receiving regimens containing T-DXd for ≥ 2L treatment or regimens containing tucatinib for ≥ 3L treatment, it is possible that TTNT and attrition rates post-2021 may have been impacted as the treatments are more efficacious than previous standard-of-care regimens prescribed between 2017 and 2021. Further research to explore the impact of the changing drug landscape on attrition rates would be insightful.

This study has several strengths; the LOT algorithm aligns with the ESMO guidelines and was standardized across centers [21], ensuring consistency. Sensitivity analyses of the robustness of the LOT algorithm performed well, with minimal variance in classification of LOTs and treatment regimens when tested under different parameters (Online resource 4**)**. For all participating centers, death data were captured from EMRs linked to local or national death registries within each country, or ascertained via manual death look-up outside the EMR, resulting in a higher complete capture of death and more accurate reporting of attrition rates, PFS and TTNT than if events were captured from EMR databases alone. Key clinical and outcome data were available for these analyses as both structured and unstructured data were collected, allowing for granularity of the clinical data. Variables such as progression events that were unlikely to be captured in the structured EMR were manually abstracted from patient notes to enable appropriate capture of the events and mitigate underreporting of progression from structured data alone. Data checks were conducted to assess the degree of missingness and the extent of outlier values for key variables at the outset of analysis, with a common data model used to harmonize the data.

This study had various limitations: heterogeneity between centers in the study and the modest sample size at each center limit interpretation of differences in patient populations attending each hospital system, clinician attitudes to and choice of treatment, access to treatments, and treatment costs. Generalizability of reasons for attrition (e.g., move to EOL care) to other European countries is constrained by the use of a small number of sites in selected European countries. Leveraging methods to ensure consistency across the centers helped minimize between-center differences in classification of attrition. Exclusive use of secondary and potentially incomplete data meant that LOTs may have been misclassified for patients who completed only part of their patient journey at the selected oncology centers. This may have played a larger role in countries with a decentralized healthcare provider structure (e.g., Germany), where patients may be treated in multiple centers, switching healthcare providers during the course of their disease. To minimize misclassification of LOT, efforts were made at each center to collect complete treatment information; patients who had clearly incomplete records were excluded. To check algorithm performance, a Medical Oncologist and an Epidemiologist reviewed a random sample of 100 patients prior to study initiation, which led to two small revisions improving algorithm performance: including the (F)EC-TPH treatment regimen and allowing for intra-class drug switching. However, as not all patients’ treatment journeys were manually reviewed with respect to algorithm performance there is a chance some misclassification was introduced. Factors associated with treatment outcomes, such as details of tumor burden, disease progression, and central nervous system metastasis, may be underreported; however, this is likely to be non-differential with respect to attrition and would not qualitatively impact results. EMR databases at some centers have limited ability to capture all reasons for stopping a treatment, which do not always reflect the precluding circumstances, potentially contributing to reasons classified as “other.”

Our definition of attrition rate excluded patients whose previous LOT was ongoing from the numerator (Fig. 1); an alternative definition that includes patients who *started* the previous LOT would have resulted in lower attrition rates and would not have accounted for patients remaining on an earlier LOT. Eastern Cooperative Oncology Group (ECOG) performance status is an important prognostic factor that was not collected from sites in this study, but may have impacted TTNT from 1 and 2L and from 2 to 3L. Additionally, TTNT2 (the time from the LOT start date to subsequent LOT initiation, death, censoring at the last known activity date at the oncology center or end of study, whichever occurred first) from 1 to 3L was not analysed in the current study, but may have provided useful reference for assessing treatment sequences in future research. During the study period the COVID-19 pandemic and subsequent lockdowns occurred, which may have impacted the ability of centers to provide palliative and anticancer therapy and consequently affected attrition rates. Finally, this research provides limited insights to establishing optimal treatment sequencing for patients with HER2 + mBC.

## Conclusions

Real-world attrition rates for patients with HER2 + mBC in Europe are not well characterized. The results we report in this patient population show that attrition rates after 1L and 2L are high and suggest that effectiveness decreases with each subsequent LOT. This highlights the importance of implementing effective drug-sequencing strategies to provide guideline-concordant care in the earliest indicated setting, given the potential loss of opportunity with each subsequent LOT. Additional strategies to improve treatment rates may include early diagnosis of progression, prehabilitation and frailty management. Further research is needed to understand reasons for attrition not captured within the current study, which may reflect differences in patient fitness, clinical practice across centers, patient preference and attitudes to treatment (which may be related to factors such as age), and financial and regulatory barriers to accessing treatments across centers.

## Supplementary Information

Below is the link to the electronic supplementary material.

Supplementary file1 (DOCX 219 KB)

## Data Availability

We will adhere to the ethical obligations for responsible sharing of data. The data that support the findings of this study are not publicly available due to patient privacy and legal restrictions. Requests for access to the de-identified data can be submitted to catherine.hogg@iqvia.com and grainne.long@astrazeneca.com for review and approval; however, requests for patient-level data cannot be granted due to legal and study ethics approval requirements.

## Abbreviations

1L: First line
2L: Second line
3L: Third line
A: Attrition
BC: Breast cancer
BMI: Body mass index
CI: Confidence interval
COVID-19: Coronavirus disease 2019
CDK4/6i: Cyclin-dependent kinase 4 and 6 inhibitor
EMR: Electronic medical record
EOL: End of life
ESMO: European Society for Medical Oncology
FU: Follow up
HER2: Human epidermal growth factor receptor 2
HR: Hormone receptor
IO: Immunotherapy
LOT: Line of therapy
mBC: Metastatic breast cancer
N: Number of patients
PFS: Progression-free survival
TTD: Time to treatment discontinuation
TTNT: Time to next treatment
