# Supplementary material for: Attrition between lines of therapy and real-world outcomes of patients with HER2-positive metastatic breast cancer in Europe: a cohort study leveraging electronic medical records
Source: Breast Cancer Res Treat. 2024 Oct 20;209(2):419–30. doi: 10.1007/s10549-024-07506-4 (PMC11785661; doi:10.1007/s10549-024-07506-4)
Supplement: Supplementary file 1 — Supplementary file1 (DOCX 219 KB) [file 10549_2024_7506_MOESM1_ESM.docx]

**Attrition between lines of therapy and real-world outcomes of patients with**
**HER2-positive metastatic breast cancer in Europe:** a cohort study leveraging electronic medical records

Paul Cottu^1^, Sue Cheeseman^2^, Peter Hall^3,4^, Achim Wöckel^5^, Christian W Scholz^6^, Emilio Bria^7,8^,
Armando Orlandi^7^, Nuria Ribelles^9^, Mahéva Vallet^3,4^, Nicolas Niklas^10^, Catherine Hogg^11^, Shivani Aggarwal^12^, Joana Moreira^13^ Markus Lucerna^14^, Simon M Collin^15^, Amanda Logue^16^, Gráinne H Long^15^

1. Université Paris Cité & Department of Medical Oncology, Institut Curie, Paris, France
2. Leeds Teaching Hospitals NHS Trust, Leeds, UK
3. University of Edinburgh, Edinburgh, UK
4. Edinburgh Cancer Centre, Western General Hospital, NHS Lothian, Edinburgh, UK
5. Department of Gynecology, University Hospital Würzburg, Würzburg, Germany
6. Department of Hematology and Oncology, Vivantes Klinikum Am Urban, Berlin, Germany
7. Unit of Oncology, Comprehensive Cancer Center, Fondazione Policlinico Universitario Agostino Gemelli IRCCS, Università Cattolica del Sacro Cuore, Rome, Italy
8. Ospedale Isola Tiberina – Gemelli Isola, Roma, Italy
9. Department of Medical Oncology, Hospital Universitario Virgen de la Victoria, IBIMA, Málaga, Spain
10. IQVIA, Frankfurt, Germany
11. IQVIA, London, UK
12. Landmark Science, Los Angeles, California, US
13. IQVIA, Lisbon, Portugal
14. Daiichi Sankyo Europe GmbH, Munich, Germany
15. Oncology Outcomes Research, Global Medical Affairs, Oncology Business Unit, AstraZeneca, Cambridge, UK
16. Medical Communications & Information, Global Medical Affairs, Oncology Business Unit, AstraZeneca, Cambridge, UK

**Corresponding author:** Paul Cottu

E-mail: paul.cottu@curie.fr, ORCiD ID: 0000-0001-6434-3932

**Journal:** Breast Cancer Research and Treatment

# Supplementary information

**Table of Contents**

[Online resource 1 3](#_Toc170297816)

[Online resource 2 4](#_Toc170297817)

[Online resource 3 5](#_Toc170297818)

[Online resource 4 6](#_Toc170297819)

[Online resource 5 7](#_Toc170297820)

[Online resource 6 8](#_Toc170297821)

[Online resource 7 10](#_Toc170297822)

[Online resource 8 11](#_Toc170297823)

[Online resource 9 12](#_Toc170297824)

[Online resource 10 13](#_Toc170297825)

[Online resource 11 14](#_Toc170297826)

[References 15](#_Toc170297827)

Online resource 1 Summary of HER2-directed agents and when they were approved by the EMA for patients with HER2+ mBC [1–8]

**T-DXd** approved for **≥2L** treatment of adult patients with unresectable or metastatic HER2+ BC who had received **one or more** HER2-directed treatments

*Based on results from the DESTINY-Breast03 trial [8]*

T-**DXd** approved for **>2L** treatment of adult patients with unresectable or metastatic HER2+ BC who had received **two or more**
HER2-directed treatments

*Based on results from the DESTINY-Breast01 trial [7]*

**T-DM1** approved for **2L** treatment of patients with advanced or metastatic HER2+ BC who had previously received trastuzumab and a taxane

*Based on results from the EMILIA trial [2]*

**2000**

**2001**

**2002**

**2003**

**2004**

**2005**

**2006**

**2007**

**2008**

**2009**

**2010**

**2011**

**2012**

**2013**

**2014**

**2015**

**2016**

**2017**

**2018**

**2019**

**2020**

**2021**

**2022**

**Tucatinib + trastuzumab + capecitabine** for
**>2L** treatment of adult patients with unresectable or metastatic HER2+ BC

*Based on results from the
HER2CLIMB trial [6]*

**Trastuzumab + docetaxel or paclitaxel** approved for the **1L** treatment of patients with HER2+ mBC

*Based on results from
Slamon DJ, et al. New Eng J Med 2001 [1]*

**Pertuzumab + trastuzumab + docetaxel** approved for **1L** treatment of adult patients with HER2+ mBC

*Based on results from the CLEOPATRA trial [3]*

**Lapatinib + capecitabine** approved for the **2L** treatment of patients with advanced or metastatic HER2+ BC who had previously received an anthracycline and a taxane, and following treatment of the patient’s metastatic disease with trastuzumab

*Based on results from Geyer CE, et al. New Eng J Med 2006 [4]*

**Lapatinib + trastuzumab** approved for the **2L** treatment of patients with
advanced or metastatic HER2+ BC previously treated with
trastuzumab + chemotherapy

*Based on results from Blackwell KL, et al. J Clin Oncol 2012 [5]*

*1L* first line, *2L* second line, *EMA*, European Medicines Agency, *HER2* human epidermal growth factor receptor 2, *mBC* metastatic breast cancer, *T-DM1* trastuzumab emtansine, *T-DXd* trastuzumab deruxtecan

Online resource 2 Study diagram [9]

Seven European hospital groups were identified from IQVIA’s oncology evidence network centers
(refer to **Online resource 3** for more details on the participating centers)

LOT algorithm was developed based on
ESMO guidelines [9]

Attrition rate algorithm was defined as
‘*the percentage of patients with no further evidence of treatment who did not receive the subsequent LOT*’

Patients with BC and metastases diagnosed between January 01, 2017 and June 30, 2021 (*n*=3304)

Patients excluded from the study:

- Presence of co-malignancies within 1 year prior to mBC diagnosis^a^
- Participation in clinical trial on or after the data of metastasis
- Incomplete treatment pathway captured in EMR
- Patient opted out of use of their data

Medical Oncologist and an Epidemiologist manually reviewed data from a sample of 100 patients for drug start/end dates,
algorithm-assigned LOTs and potential misclassification of LOTs

EMR data from eligible patients with HER2+ mBC were collected (*n*=496)

LOT algorithm was further tested by two sensitivity analyses
(refer to **Online resource 4** for more details on the sensitivity analyses)

Structured EMR data and manually abstracted unstructured data were curated, cleaned, and quality checked

Attrition rate algorithm was applied to the cohort

*BC* breast cancer*, EMR* electronic medical record*, ESMO* European Society of Medical Oncology, *HER2* human epidermal growth factor receptor 2*, LOT* line of therapy*, mBC* metastatic breast cancer

^a^Excluding non-melanoma skin cancer and in situ or benign neoplasms

Online resource 3 Details about each hospital in the RESTORE study

Participating centers included Institut Curie, a comprehensive cancer center treating approximately 17,000 oncology patients per annum (national and international), including approximately 4,000 new patients with breast cancer annually, in Paris, Saint-Cloud and Orsay, France; one hospital from the Vivantes Hospital Group, Germany’s largest municipal hospital system that treats over 500,000 patients annually; University Hospital Würzburg (Germany), comprising 19 clinical centers, three policlinics, and four clinical institutes that together treat more than 300,000 patients per year; NHS Lothian (UK), a teaching hospital treating approximately 20,000 patients annually; Leeds Teaching Hospitals Trust (UK), a major regional and national NHS cancer center providing secondary and tertiary care to over 750,000 and five million patients, respectively; Fondazione Policlinico Universitario Agostino Gemelli, IRCCS (Rome, Italy), a referral hospital treating over 50,000 Italian and international patients every year; and the Unidad de Gestión Clínica Intercentros (UCGI) Oncology, comprised of two referral centers covering over one million inhabitants of Málaga, Spain.

Online resource 4 Methodology and results for validation of the LOT algorithm

- For validation of the LOT algorithm, a random sample of 100 metastatic breast cancer (mBC) patients’ profiles from hospitals in the UK, Germany, and France were manually reviewed by a Medical Oncologist and an Epidemiologist to evaluate drug start/end dates and algorithm-assigned lines of therapy (LOTs), and identify any potential misclassification of LOT and treatment regimens
- This led to two small revisions improving algorithm performance: including the (F)EC-TPH regimen and allowing for intra-class drug switching
- Following manual review of sample mBC patient profiles, two sensitivity analyses were undertaken to further validate the LOT algorithm
- Five time windows, noted below, were altered using data from UK, Germany, and France centers to assess the impact on LOTs and regimens assigned by the algorithm

| Time windows tested in the sensitivity analyses to validate the LOT algorithm | | | |
| --- | --- | --- | --- |
| Time window | Base case | Sensitivity analysis 1 | Sensitivity analysis 2 |
| Window for allowable treatment prior to start of metastasis | 14 days | 21 days | 7 days |
| Window for drugs to be considered part of a regimen, from the start of the LOT | 30 days | 45 days | 28 days |
| Window for treatments initiated prior to metastasis continuing past the date of metastasis | 60 days | 7 days | 90 days |
| Window for treatments after the date of metastasis if only adjuvant hormonal therapy is present in the 14 days prior to the date of metastasis | 90 days | 60 days | 120 days |
| Treatment holiday window | 365 days | 180 days | 270 days |

*LOT* line of therapy

- The sensitivity analysis results indicated that the LOT algorithm was robust, as the number of 1L treatments did not vary for 1L and varied by <3.5% for 2L and 3L
- Additionally, the most frequent regimens seen in over half of patients did not change and there were only minor differences in the remaining regimens

| **Online resource 5** Packages and workstreams used for statistical analysis and harmonization by the statistical tool R v4.1.3 | |
| --- | --- |
| Packages used for statistical analysis | Packages used for data transformations / management |
| - survival 3.2-13 - survminer 0.4.9 - iqvir 2.0.2 | - ggpubr 0.6.0 - ggplot2 3.4.2 - readxl 1.4.2 - lubridate 1.9.2 - stringr 1.5.0 - openxlsx 4.2.5.2 - tidyr 1.3.0 - dplyr 1.1.2 - magrittr 2.0.3 - data.table 1.14.8 - haven 2.5.2 |

| **Online resource 6** Patient demographics and clinical characteristics by country and hormone receptor status | | | | | | | | |
| --- | --- | --- | --- | --- | --- | --- | --- | --- |
|  | Overall  (N=496) | France  (*n* =115) | Germany  (*n* =84) | Italy  (*n* =105) | Spain  (*n* =56) | UK  (*n*=136) | HR+  (*n* =328) | HR-  (*n*=163) |
| Median age at initial diagnosis (IQR), years | 56.0 (47.0–70.0) | 54.0 (45.0–68.0) | 66.0 (55.0–73.0) | 53.0 (45.0–61.0) | 54.0 (47.0–68.0) | 59.0 (48.0–73.0) | 57.0 (7.0–70.0) | 55.0 (46.0–70.0) |
| Median age at mBC diagnosis (IQR), years | 59.0 (49.0–72.0) | 56.0 (46.0–71.0) | 67.5 (57.0–76.0) | 56.0 (47.0–63.0) | 55.5 (49.0–68.0) | 61.5 (49.0–73.0) | 60.0 (50.0–72.0) | 57.0 (48.0–71.0) |
| Median BMI (IQR), kg/m^2^ | 25.7 (22.5–29.7) | 24.7 (22.0–28.6) | 26.0 (23.0–29.1) | 24.2 (22.1–28.8) | 26.3 (23.6–30.1) | 28.6 (24.3–31.9) | 26.0 (22.5–30.3) | 25.0 (22.4–28.7) |
| Postmenopausal status at mBC diagnosis, *n* (%) | 293 (59.1) | 52 (45.2) | 57 (67.9) | 45 (42.9) | 40 (71.4) | 99 (72.8) | 196 (59.8) | 96 (58.9) |
| Current smoker, *n* (%) | 75 (15.1) | 24 (20.9) | 6 (7.1) | 15 (14.3) | 13 (23.2) | 17 (12.5) | 46 (14.0) | 29 (17.8) |
| Primary tumor type, *n* (%) |  |  |  |  |  |  |  |  |
| Invasive ductal carcinoma | 424 (85.5) | 104 (90.4) | 70 (83.3) | 77 (73.3) | 54 (96.4) | 119 (87.5) | 277 (84.5) | 144 (88.3) |
| Invasive lobular carcinoma | 22 (4.4) | 6 (5.2) | 5 (6.0) | 8 (7.6) | 2 (3.6) | 1 (0.7) | 15 (4.6) | 7 (4.3) |
| Other | 29 (5.9) | 5 (4.4) | 4 (4.8) | 5 (4.8) | 0 (0.0) | 15 (11.0) | 22 (6.7) | 7 (4.3) |
| Unknown | 17 (3.4) | 0 (0.0) | 1 (1.2) | 15 (14.3) | 0 (0.0) | 1 (0.7) | 12 (3.7) | 4 (2.5) |
| Missing | 4 (0.8) | 0 (0.0) | 4 (4.8) | 0 (0.0) | 0 (0.0) | 0 (0.0) | 2 (0.6) | 1 (0.6) |
| Metastatic sites at BC diagnosis, *n* (%) |  |  |  |  |  |  |  |  |
| <4 | 360 (72.6) | 83 (72.2) | 61 (72.6) | 76 (72.4) | 48 (85.7) | 92 (67.6) | 244 (74.4) | 112 (68.7) |
| ≥4 | 136 (27.4) | 32 (27.8) | 23 (27.4) | 29 (27.6) | 8 (14.3) | 44 (32.4) | 84 (25.6) | 51 (31.3) |
| Metastatic location, *n* (%)^a^ |  |  |  |  |  |  |  |  |
| Local/breast | 146 (29.4) | 38 (33.0) | 34 (40.5) | 16 (15.2) | 3(5.4) | 55 (40.4) | 95 (29.0) | 50 (30.7) |
| Brain | 151 (30.4) | 19 (16.5) | 22 (26.2) | 47 (44.8) | 19 (33.9) | 44 (32.4) | 85 (25.9) | 64 (39.3) |
| Bone | 285 (57.5) | 69 (60.0) | 54 (64.3) | 58 (55.2) | 35 (62.5) | 69 (50.7) | 202 (61.6) | 82 (50.3) |
| Lung | 193 (38.9) | 40 (34.8) | 36 (42.9) | 41 (39.0) | 14 (25.0) | 62 (45.6) | 122 (37.2) | 66 (40.5) |
| Liver | 229 (46.2) | 48 (41.7) | 44 (52.4) | 47 (44.8) | 20 (35.7) | 70 (51.5) | 152 (46.3) | 75 (46.0) |
| Lymph nodes | 258 (52.0) | 71 (61.7) | 39 (46.4) | 55 (52.4) | 22 (39.3) | 71 (52.2) | 159 (48.5) | 96 (58.9) |
| Other | 111 (22.4) | 32 (27.8) | 28 (33.3) | 11 (10.5) | 19 (33.9) | 21 (15.4) | 74 (22.6) | 36 (22.1) |
| Stage IV de-novo disease at initial BC diagnosis, *n* (%) | 302 (60.9) | 76 (66.1) | 48 (57.1) | 58 (55.2) | 30 (53.6) | 90 (66.2) | 200 (61.0) | 101 (62.0) |
| Grading, *n* (%) |  |  |  |  |  |  |  |  |
| Grade 1 | 3 (0.6) | 3 (2.6) | 0 (0.0) | 0 (0.0) | 0 (0.0) | 0 (0.0) | 3 (0.9) | 0 (0.0) |
| Grade 2 | 157 (31.7) | 37 (32.2) | 25 (29.8) | 23 (21.9) | 14 (25.0) | 58 (42.6) | 105 (32.0) | 51 (31.3) |
| Grade 3 | 289 (58.3) | 75 (65.2) | 50 (59.5) | 66 (62.9) | 21 (37.5) | 77 (56.6) | 194 (59.1) | 93 (57.1) |
| Unknown | 42 (8.5) | 0 (0.0) | 4 (4.8) | 16 (15.2) | 21 (37.5) | 1 (0.7) | 24 (7.3) | 17 (10.4) |
| Missing | 5 (1.0) | 0 (0.0) | 5 (6.0) | 0 (0.0) | 0 (0.0) | 0 (0.0) | 2 (0.6) | 2 (1.2) |
| Stage at initial BC diagnosis, *n* (%) |  |  |  |  |  |  |  |  |
| 0/I/II/III | 169 (34.1) | 35 (30.4) | 30 (35.7) | 34 (32.4) | 26 (46.4) | 44 (32.4) | 110 (33.5) | 57 (35.0) |
| IV | 302 (60.9) | 76 (66.1) | 48 (57.1) | 58 (55.2) | 30 (53.6) | 90 (66.2) | 200 (61.0) | 101 (62.0) |
| Unknown | 18 (3.6) | 2 (1.7) | 1 (1.2) | 13 (12.4) | 0 (0.0) | 2 (1.5) | 13 (4.0) | 4 (2.5) |
| Missing | 7 (1.4) | 2 (1.7) | 5 (6.0) | 0 (0.0) | 0 (0.0) | 0 (0.0) | 5 (1.5) | 1 (0.6) |
| Total number of LOTs per patient, *n* (%)^b^ |  |  |  |  |  |  |  |  |
| 0 | 34 (6.9) | 0 | 5 (6.0) | 5 (4.8) | 1 (1.8) | 23 (16.9) | 18 (5.5) | 14 (8.6) |
| 1 | 208 (41.9) | 44 (38.3) | 44 (52.4) | 47 (44.8) | 26 (46.4) | 47 (34.6) | 133 (40.5) | 74 (45.4) |
| 2 | 131 (26.4) | 37 (32.2) | 20 (23.8) | 33 (31.4) | 11 (19.6) | 30 (22.1) | 94 (28.7) | 35 (21.5) |
| 3 | 61 (12.3) | 17 (14.8) | 6 (7.1) | 11 (10.5) | 11 (19.6) | 16 (11.8) | 40 (12.2) | 21 (12.9) |
| Median duration of FU (IQR), months | 41.1 (22.4–52.8) | 38.8 (21.3–51.4) | 24.4 (12.1–44.5) | 45.4 (29.4–56.3) | 40.9 (20.2–56.5) | 44.0 (27.8–54.0) | 41.4 (22.4–54.0) | 39.9 (22.2–51.3) |

*BMI* body mass index, *FU* follow up, *HR* hormone receptor, *IQR* interquartile range, *LOT* line of therapy, *mBC* metastatic breast cancer

^a^Patients may belong to >1 category

^b^*n*=62 patients received ≥4 LOTs per patient

Online resource 7 Attrition rates from 1L to 2L and 2L to 3L, stratified by country and HR status^a^

1L to 2L

2L to 3L

|  | **OVERALL** | | **HR+** | | **HR–** | | **France** | | **Germany** | | **Italy** | | **Spain** | | **UK** | |
| --- | --- | --- | --- | --- | --- | --- | --- | --- | --- | --- | --- | --- | --- | --- | --- | --- |
| **95% CI** | 25.0, 34.6 | 27.5, 41.5 | 21.1, 32.6 | 24.9, 42.1 | 27.2, 45.3 | 23.7, 48.7 | 7.7,  23.9 | 14.3, 41.1 | 30.0, 55.9 | 23.4, 63.1 | 21.1, 42.7 | 31.0,  64.2 | 17.6, 47.1 | 5.2,  40.3 | 23.5, 42.9 | 22.2, 48.6 |
| **n/N** | 107/361 | 64/187 | 64/241 | 41/124 | 42/117 | 22/62 | 12/83 | 12/46 | 26/61 | 11/26 | 24/77 | 18/38 | 13/42 | 4/22 | 32/98 | 19/55 |

*1L* first line, *2L* second line, *3L* third line, *CI* confidence interval, *HR* hormone receptor

^a^France, one hospital; Germany, two hospital groups; Italy, one hospital; Spain, one hospital group; UK, two hospital groups

| Online resource 8 “Other” reasons for attrition (pooled)^a^ | | |
| --- | --- | --- |
|  | 1L (*n*=32) | 2L (*n*=26) |
| Died | 12 | 6 |
| Move to end-of-life palliative care without death | 0 | 5 |
| Loss to FU | 2 | 7 |
| Other | 18 | 8 |
| Other known^b^ | 7 | 3 |
| Missing | 10 | 4 |
| Progression | 1 | 1 |

*1L* first line, *2L* second line, *FU* follow up

^a^This sensitivity analysis examined the impact of extending the time window for categorizing reason for attrition within ‘other’ from within 30 days after treatment discontinuation to >30 days (and up to the end of the study period) in terms of death, move to end-of-life palliative care, or loss to FU

^b^‘Other known’ may include patient choice, protocol or physician choice, and palliative treatment, which were not collected at all sites during this study

| **Online resource 9** Univariate Cox proportional hazard regression models for 1L TTNT | | | |
| --- | --- | --- | --- |
| Univariate association | | Hazard ratio (95% CI) | *P*-value |
| Age (years) | <50 | Reference |  |
|  | 50–59 | 0.98 (0.72–1.33) | 0.901 |
|  | 60–69 | 0.83 (0.62– 2.11) | 0.213 |
|  | ≥70 | 1.45 (0.96–2.19) | 0.079 |
| Number of sites  of metastasis | ≥2 | Reference |  |
|  | 1 | 0.76 (0.57–1.01) | 0.056 |
| Brain metastases | Yes | Reference |  |
|  | No | 1.06 (0.82–1.37) | 0.655 |
| Liver metastases | Yes | Reference |  |
|  | No | 0.84 (0.67–1.06) | 0.149 |
| HR status | Positive | Reference |  |
|  | Negative | 0.97 (0.76–1.24) | 0.826 |
| Time from initial diagnosis to metastases | ≥24 months | Reference |  |
|  | <24 months | 1.03 (0.71–1.49) | 0.896 |
|  | 0 months | 0.87 (0.66–1.14) | 0.308 |
| Stage at BC diagnosis | Stages 0–I | Reference |  |
|  | Stage II | 1.01 (0.60–1.68) | 0.979 |
|  | Stage III | 1.47 (0.87–2.49) | 0.151 |
|  | Stage IV | 0.96 (0.62–1.50) | 0.869 |
|  | Unknown | 1.02 (0.53–1.94) | 0.954 |
| Menopausal status at  mBC diagnosis | Postmenopausal | Reference |  |
|  | Premenopausal | 1.11 (0.85–1.44) | 0.446 |
|  | Unknown | 0.93 (0.66–1.29) | 0.650 |
| Grade | Grade 1–2 | Reference |  |
|  | Grade 3 | 0.74 (0.58–0.94) | 0.014 |
|  | Unknown | 0.64 (0.42–0.99) | 0.044 |
| Histology | Invasive ductal carcinoma | Reference |  |
|  | Invasive lobular carcinoma or other | 0.86 (0.59–1.25) | 0.427 |

*1L* first line, *BC* breast cancer, *CI* confidence interval, *HR* hormone receptor, *mBC* metastatic breast cancer, *TTNT* time to next treatment

| **Online resource 10** Univariate Cox proportional hazard regression models for 2L TTNT | | | |
| --- | --- | --- | --- |
| Univariate association | | Hazard ratio (95% CI) | *P*-value |
| Age (years) | <50 | Reference |  |
|  | 50–59 | 1.29 (0.83–2.00) | 0.250 |
|  | 60–69 | 1.21 (0.78–1.88) | 0.390 |
|  | ≥70 | 1.52 (0.82–2.83) | 0.182 |
| Number of sites of metastasis | ≥2 | Reference |  |
|  | 1 | 0.54 (0.33–0.88) | 0.013 |
| Brain metastases | Yes | Reference |  |
|  | No | 0.92 (0.63–1.33) | 0.653 |
| Liver metastases | Yes | Reference |  |
|  | No | 0.72 (0.52–1.01) | 0.057 |
| HR status | Positive | Reference |  |
|  | Negative | 1.29 (0.90–1.84) | 0.165 |
| Time from initial diagnosis to metastases | ≥24 months | Reference |  |
|  | <24 months | 1.07 (0.62–1.83) | 0.810 |
|  | 0 months | 0.74 (0.51–1.09) | 0.131 |
| Stage at BC diagnosis | Stages 0–III | Reference |  |
|  | Stage IV | 0.72 (0.51–1.04) | 0.078 |
| Menopausal status at mBC diagnosis | Postmenopausal | Reference |  |
|  | Premenopausal | 0.66 (0.44–0.97) | 0.033 |
|  | Unknown | 0.66 (0.41–1.08) | 0.101 |
| Grade | Grade 1–2 | Reference |  |
|  | Grade 3 | 0.83 (0.58–1.17) | 0.288 |
| Histology | Invasive ductal carcinoma | Reference |  |
|  | Invasive lobular carcinoma or other | 0.94 (0.52–1.70) | 0.837 |

*2L* second line, *BC* breast cancer, *CI* confidence interval, *HR* hormone receptor,
*mBC* metastatic breast cancer, *TTNT* time to next treatment

Online resource 11 PFS from 1L to 3L


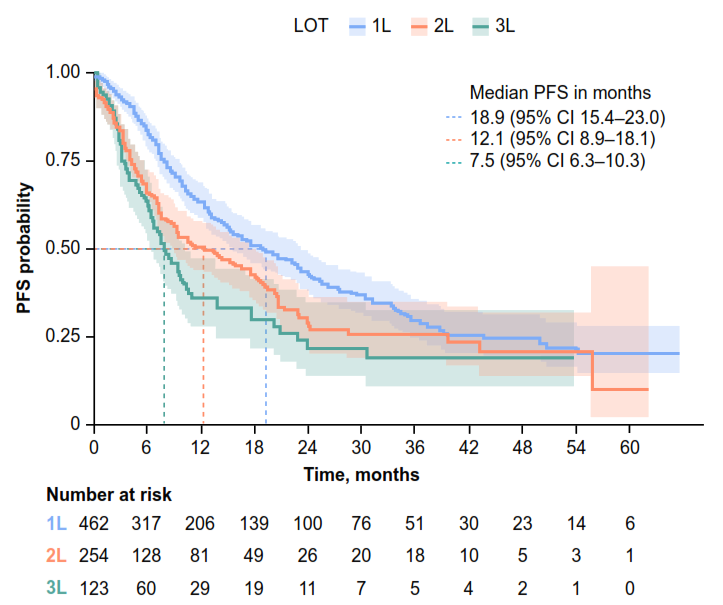
 *1L* first line, *2L* second line, *3L* third line, *CI* confidence interval, *LOT* line of therapy, *PFS* progression-free survival

# References

1. Slamon DJ, Leyland-Jones B, Shak S et al (2001) Use of chemotherapy plus a monoclonal antibody against HER2 for metastatic breast cancer that overexpresses HER2. N Engl J Med 344:783–792. https://doi.org/10.1056/NEJM200103153441101

2. Verma S, Miles D, Gianni L et al (2012) Trastuzumab emtansine for HER2-positive advanced breast cancer. N Engl J Med 367:1783–1791. https://doi.org/10.1056/NEJMoa1209124

3. Baselga J, Cortés J, Kim S-B et al (2012) Pertuzumab plus trastuzumab plus docetaxel for metastatic breast cancer. N Engl J Med 366:109–119. https://doi.org/10.1056/NEJMoa1113216

4. Geyer CE, Forster J, Lindquist D et al (2006) Lapatinib plus capecitabine for HER2-positive advanced breast cancer. N Engl J Med 355:2733–2743. https://doi.org/10.1056/NEJMoa064320

5. Blackwell KL, Burstein HJ, Storniolo AM et al (2012) Overall survival benefit with lapatinib in combination with trastuzumab for patients with human epidermal growth factor receptor 2-positive metastatic breast cancer: final results from the EGF104900 Study. J Clin Oncol 30:2585–2592. https://doi.org/10.1200/JCO.2011.35.6725

6. Murthy RK, Loi S, Okines A et al (2020) Tucatinib, trastuzumab, and capecitabine for HER2-positive metastatic breast cancer. N Engl J Med 382:597–609. https://doi.org/10.1056/NEJMoa1914609

7. Modi S, Saura C, Yamashita T et al (2020) Trastuzumab deruxtecan in previously treated HER2-positive breast cancer. N Engl J Med 382:610–621. https://doi.org/10.1056/NEJMoa1914510

8. Cortés J, Kim S-B, Chung W-P et al (2022) Trastuzumab deruxtecan versus trastuzumab emtansine for breast cancer. N Engl J Med 386:1143–1154. https://doi.org/10.1056/NEJMoa2115022

9. Gennari A, André F, Barrios CH et al (2021) ESMO Clinical Practice Guideline for the diagnosis, staging and treatment of patients with metastatic breast cancer. Ann Oncol 32:1475–1495. https://doi.org/10.1016/j.annonc.2021.09.019
